# The Actin/Spectrin Membrane-Associated Periodic Skeleton in Neurons

**DOI:** 10.3389/fnsyn.2018.00010

**Published:** 2018-05-23

**Authors:** Nicolas Unsain, Fernando D. Stefani, Alfredo Cáceres

**Affiliations:** ^1^Instituto de Investigación Médica Mercedes y Martín Ferreyra (INIMEC), Consejo Nacional de Investigaciones Científicas y Técnicas (CONICET), Córdoba, Argentina; ^2^Universidad Nacional de Córdoba, Córdoba, Argentina; ^3^Instituto Universitario Ciencias Biomédicas de Córdoba (IUCBC), Córdoba, Argentina; ^4^Centro de Investigaciones en Bionanociencias (CIBION), Consejo Nacional de Investigaciones Científicas y Técnicas (CONICET), Buenos Aires, Argentina; ^5^Departamento de Física, Facultad de Ciencias Exactas y Naturales, Universidad de Buenos Aires, Buenos Aires, Argentina

**Keywords:** actin, spectrin, axon, dendrites, cytoskeleton, super resolution microscopy, fluorescence nanoscopy

## Abstract

Neurons are the most asymmetric cell types, with their axons commonly extending over lengths that are thousand times longer than the diameter of the cell soma. Fluorescence nanoscopy has recently unveiled that actin, spectrin and accompanying proteins form a membrane-associated periodic skeleton (MPS) that is ubiquitously present in mature axons from all neuronal types evaluated so far. The MPS is a regular supramolecular protein structure consisting of actin “rings” separated by spectrin tetramer “spacers”. Although the MPS is best organized in axons, it is also present in dendrites, dendritic spine necks and thin cellular extensions of non-neuronal cells such as oligodendrocytes and microglia. The unique organization of the actin/spectrin skeleton has raised the hypothesis that it might serve to support the extreme physical and structural conditions that axons must resist during the lifespan of an organism. Another plausible function of the MPS consists of membrane compartmentalization and subsequent organization of protein domains. This review focuses on what we know so far about the structure of the MPS in different neuronal subdomains, its dynamics and the emerging evidence of its impact in axonal biology.

## Introduction

Plasma membrane domain specialization is determinant for key cellular activities such as adhesion, signaling, membrane excitability, endo/exocytosis and stress resistance, among others. A set of filamentous proteins have emerged early in evolution to form and maintain these domains; they are organized in a scaffold known as the actin/spectrin-based membrane skeleton, which is located at the inner surface of plasma membranes (Bennett and Baines, 2001; Baines, 2010).

Most of what we know about this skeleton comes from research characterizing the organization of the erythrocyte membrane-cortical cytoskeleton (EMCC). The EMCC is responsible for the remarkable mechanical properties of the erythrocyte, including its resistance to high shear stress and rapid changes in shape. The vital importance of the EMCC is evidenced by the fact that mutations affecting its constituents cause hereditary hemolytic anemia (Delaunay, 2007).

**Figure 1:**
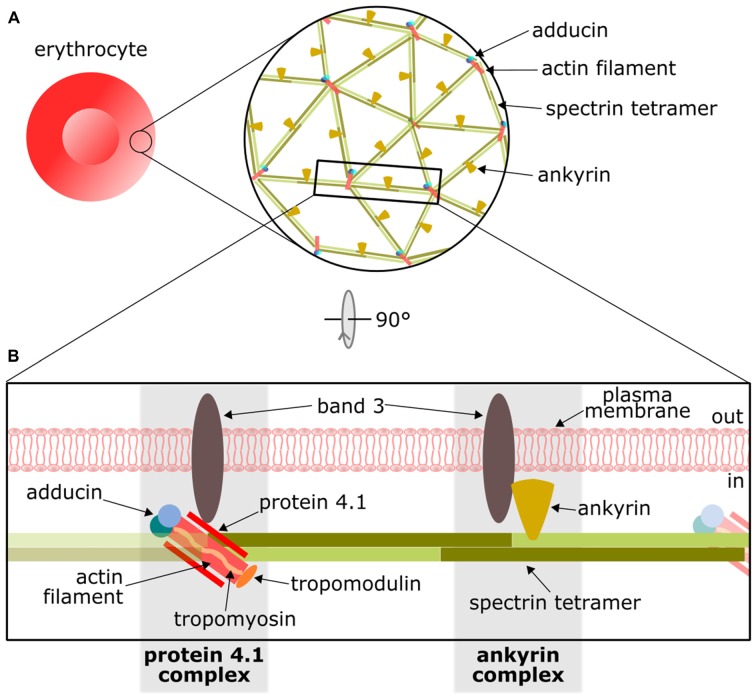
Overview of the actin/spectrin skeleton of erythrocytes. **(A)** Basic arrangement of the actin/spectrin skeleton underlying the erythrocyte membrane. **(B)** Schematic representation of the principal components of the erythrocyte membrane-cortical cytoskeleton (EMCC) and its attachments to the plasma membrane by means of the protein 4.1 and ankyrin complexes. For further details on this structure please refer to other reviews (Baines, 2010; Lux, 2016).

The detailed knowledge of the EMCC can be used as a template for studying and interpreting the distinctive cortical cytoskeleton in neurons. The two-dimensional (2D) organization of the EMCC was definitively demonstrated in the mid-1980s using negative staining electron microscopy (Byers and Branton, 1985; Shen et al., 1986), which together with additional biochemical work established a model with a repetitive 2D structure consisting of short, ~37 nm long actin filaments (composed of 12 to 14 monomers) that coordinate 5 or 6 αI/βI-spectrin tetramers (Figure 1A). Accessory proteins, namely protein 4.1 and dimers of α/β-adducin, further stabilize the actin-spectrin interaction. The whole structure is attached to the plasma membrane through two protein complexes that interact with the anion transport protein Band 3. At the actin-spectrin junction, the protein 4.1 complex mediates the interaction with Band 3, whereas a second link to the membrane is formed through an ankyrin* complex* close to the middle point of the spectrin tetramer (Figure 1B). The principal components of the EMCC are also found in other cell types suggesting it provides essential biological functions (Baines, 2009, 2010).

In the nervous system, spectrins have key roles in membrane domain organization. Experimental deletion of αII-, βII-, βIII- and βIV-spectrin forms, as well as mutations found in human patients, induce drastic phenotypic defects in the nervous system, mostly related to mislocalization of neurotransmitter receptors or components of Ranvier nodes or affecting the formation of the axon initial segment (AIS; Parkinson et al., 2001; Komada and Soriano, 2002; Ikeda et al., 2006; Zhang et al., 2013; Huang et al., 2017).

## Organization of the Membrane-Associated Periodic Skeleton

**Figure 2:**
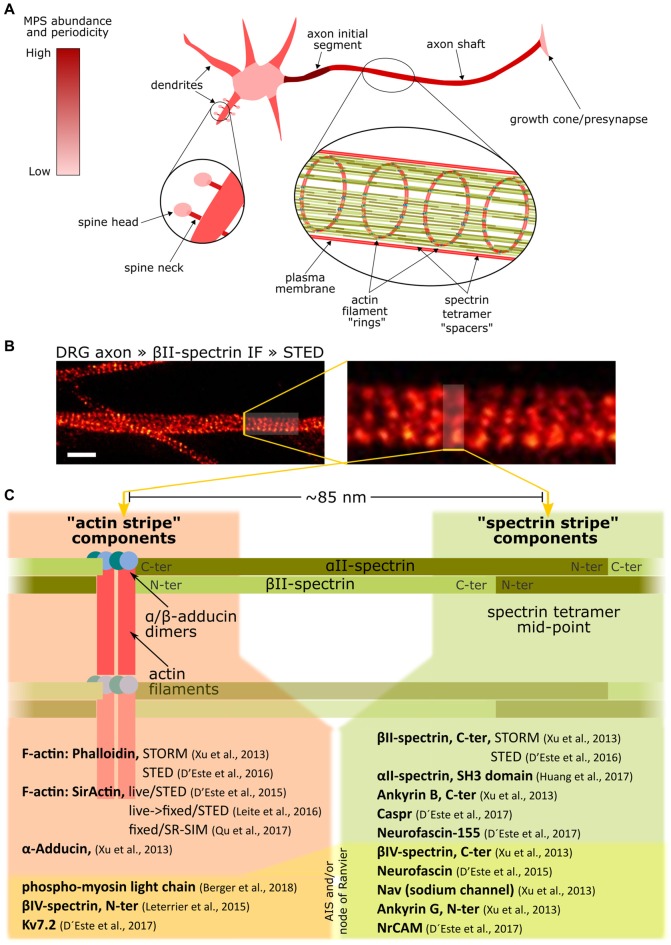
Overview of the membrane-associated periodic skeleton (MPS) of neurons and its associated proteins. **(A)** The MPS abundance and organization in different domains of a neuron, from being robust and well organized in the axon initial segment to being completely absent in the cell soma. **(B)** Axon shafts from sensory neurons in culture, stained against βII-spectrin and imaged by Stimulated Emission Depletion microscopy (STED) (Unsain et al., 2018), reveals the MPS. Scale bar 1 μm. **(C)** List of proteins arranged with a ~190 nm periodicity in axons, indicating their location with respect to the alternating actin and spectrin “stripes.” Note that the same protein can have one end in one *stripe*, and the other in the other *stripe*, as in the case of spectrins. Also, some proteins can have one of its ends interacting with the MPS and thus being periodically arranged, but have the other end freely extending out of the MPS, and thus showing no periodicity. Such is the case of ankyrin G, which has its N-terminus periodically distributed, but not its C-terminus. The components on the bottom of the list under yellow shading are exclusively found at the AIS and/or at Ranvier nodes.

In 2013, a seminal study using stochastic optical reconstruction microscopy (STORM) revealed the nanoscale organization of the actin-spectrin skeleton in axons as a periodic arrangement of F-actin rings separated by ~190 nm spectrin tetramer spacers (Xu et al., 2013), now referred to as the membrane-associated periodical skeleton (MPS, Figure 2). The resemblance to the EMCC components, together with the distance between F-actin rings equivalent to the size of a stretched spectrin tetramer, supported the conception of a structural working model of the MPS, which has been corroborated and improved by others since then: the MPS is composed of numerous short actin filaments organized in ring-like structures transverse to the axon, and separated by various αII/βII-spectrin tetramers extended along the axon (Figure 2).

A number of complementary fluorescence nanoscopy studies have revealed that the MPS is ubiquitously present in mature axons and, to a less extent, in dendrites from all neuronal types evaluated so far (Xu et al., 2013; D’Este et al., 2016; He et al., 2016). Notably, the MPS has been also observed in brain slices (Xu et al., 2013; D’Este et al., 2015) and in living cells using either permeable F-actin probes (D’Este et al., 2015) or tagged βII-spectrin expression (Zhong et al., 2014).

The MPS is also present at the AIS and Ranvier nodes, and “markers” of these domains, such as ankyrin G, βIV-spectrin, neurofascin and Nav channels organize periodically (Leterrier et al., 2015; D’Este et al., 2017). Interestingly, glial membranes at the juxtaparanode also contain proteins organized periodically (D’Este et al., 2017), probably arranged by their interaction to periodical protein lattices in the axon.

In cultured neurons, the MPS has been detected soon after axon outgrowth, but almost exclusively in regions proximal to the cell body. Over time, the periodical organization spreads distally, and by 12 days *in vitro* (DIV) the MPS can be found along the entire axonal length (Zhong et al., 2014). This proximal to distal developmental pattern awaits confirmation using unbiased sampling approaches and quantitative analysis of the MPS (Barabas et al., 2017; Unsain et al., 2018). Interestingly, the first proteins to show such a periodic arrangement during axonal growth are F-actin and βII-spectrin. αII-spectrin distributes periodically in the mature axon and seems to be the partner of βII- and βIV-spectrins in their respective tetramers (Huang et al., 2017).

Adducin is a barbed-end capping protein known to stabilize actin filaments and to prevent further incorporation of monomers; interestingly, α-adducin deletion results in actin “rings” with increased diameter (Leite et al., 2016). Adducin is found with multiple copies per ring, suggesting that each actin “ring” might be composed of several short filaments. In favor of this, platinum replica electron microscopy (PREM) revealed the existence of short actin filaments at the AIS with lengths similar to those found at the EMCC (Jones et al., 2014). Interestingly, the incorporation of adducin into the MPS is a late event occurring only after the establishment of the lattice along the axon (Zhong et al., 2014).

Current evidence suggests that the minimal components required for organizing the periodical lattice of the MPS are F-actin and spectrins, because pharmacological depolymerization of F-actin breaks the periodicity of spectrins, and βII-spectrin knock down affects F-actin periodic distribution (Xu et al., 2013; Zhong et al., 2014). Adducin, although found in the mature MPS arrangement, is not likely necessary to form the MPS, since adducin null neurons can build an MPS (Leite et al., 2016; Qu et al., 2017) and the MPS of immature axons lack adducin (Zhong et al., 2014). Instead, adducin may help in keeping F-actin filaments at a fixed length and to increase the stability of the actin-spectrin interaction, as shown for erythrocytes (Gardner and Bennett, 1987). The idea that adducin is important for the structural stability of the MPS is also supported by the fact that adducin null neurons present a decreased fraction of axonal segments with regular MPS (Qu et al., 2017).

The MPS is not fully regular along a single axon, but displays different degrees of organization and may even be absent in short segments (Barabas et al., 2017; Qu et al., 2017). Whether this irregularity reflects a meaningful biological design remains to be established, and highlights the need to determine MPS abundance (fraction of an axon/dendrite with MPS) and regularity in an unbiased and quantitative manner. Different approaches have been used to tackle these challenges, such as quantifying autocorrelation amplitudes (Zhong et al., 2014), sinusoid fit to selected regions (Leterrier et al., 2015) and computing the two-dimensional Pearson correlation against a predefined periodic pattern (Barabas et al., 2017; Unsain et al., 2018). Also, for an unbiased and high volume sampling of neurites, an open-source software was developed that integrates batch analysis, using an automated and unbiased interrogation of neurite segments by two-dimensional Pearson correlation against a modeled MPS (Barabas et al., 2017).

The lower abundance and organization of the MPS in dendrites compared to axons (D’Este et al., 2015, 2016) cannot be explained by their different diameters (Han et al., 2017). Instead, the reason may be the lower levels of βII-spectrin in dendrites. Experiments where the level of βII-spectrin was increased in dendrites resulted in a significant improvement of their MPS organization (Zhong et al., 2014). It is worth noting that under these conditions, dendrites maintain their molecular identity. On the other hand, the most common actin/spectrin organization of the somato-dendritic compartment seems to be a 2D lattice similar to the EMCC (Han et al., 2017).

Dendritic spines are specialized structures harboring most excitatory synapses. Changes in synaptic strength correlate with modifications of spine shape, which tightly depends on the organization of the underlying actin cytoskeleton. Interestingly, dendritic spines necks, but not the head, show a periodical organization of actin and spectrin (Bär et al., 2016; He et al., 2016; Sidenstein et al., 2016); the periodical lattice can be found even if the dendritic shaft from which it sprouts lacks a clear MPS. By contrast, current evidence suggests that the MPS cannot form at either side of the synapse, probably due to the high levels of synaptic proteins. For example, the AIS receives axo-axonic GABAergic synapses and at these postsynaptic sites the MPS is disrupted (D’Este et al., 2015). Also, the MPS is interrupted at presynaptic elements of *en-passant* synapses (He et al., 2016; Sidenstein et al., 2016).

## Dynamics of the MPS

The actin filament (F-actin) is a dynamic polymer, constantly incorporating and releasing monomers (G-actin). Incorporation of monomers occurs preferentially at the plus (*barbed*) end, while monomer release is more frequent at the opposite (minus) end. A variety of actin binding proteins, in turn, can organize F-actin into higher order arrangements and regulate their interaction to different cellular structures (Winder and Ayscough, 2005). Drugs affecting the actin cytoskeleton are widely used tools to estimate the impact of disturbing F-actin dynamics in a given cellular structure. Interestingly, the response of the MPS to actin-depolymerizing drugs such as cytochalasin D and latrunculin varies depending on the neuronal region. In the distal axon, doses of latrunculin that are sufficient to affect filopodial or intra-axonal longitudinal actin filaments do not disrupt the MPS; higher doses do. On the other hand, cytochalasin D affects the MPS at lower doses and faster than latrunculin, while still affecting other F-actin arrangements in the axon (Xu et al., 2013; Qu et al., 2017). In contrast, the MPS of the AIS is particularly resistant to high doses of both cytochalasin D and latrunculin (Leterrier et al., 2015). To correctly interpret these results it is important to consider the different modes of action of latrunculins and cytochalasins. Latrunculins bind exclusively to actin monomers, preventing their incorporation into filaments (Peterson and Mitchison, 2002), while cytochalasin D also caps the barbed end of actin filaments, decreasing both addition and release of monomers at this end (Peterson and Mitchison, 2002; Scherlach et al., 2010). On the other hand, both actin depolymerizing drugs have a greater effect on the MPS of immature axons than of mature ones, which correlates with the later incorporation of the actin-capping protein α-adducin (Zhong et al., 2014; Qu et al., 2017) suggesting that it may protect the barbed-end from cytochalasin D activity.

These actin-depolymerizing drugs may not target actin filaments that are part of the MPS. Instead, they may affect a pool of newly synthetized short polymers that are subsequently exchanged within the MPS. This latter possibility is supported by experiments assessing MPS abundance in fly axons, which have shown that deletion of the actin elongating factor profilin does not affect MPS abundance but deletion of actin nucleators (like formin and Arp2/3) decreases MPS abundance (Qu et al., 2017). These results support the hypothesis that the actin rings of the MPS are composed of short actin filaments, and that nucleation of new actin filaments would be more important than their growth for MPS stability.

Fluorescence recovery after photobleaching (FRAP) and live-cell STORM experiments with tagged βII-spectrin have revealed that spectrin is relatively stable in the MPS (30 min timeframe) and that the MPS structure itself may not move along the axon (6 min timeframe; Zhong et al., 2014). In support of the observed low turnover of spectrin at the MPS, mathematical modeling has suggested that tetramer exchange would be difficult in an already formed MPS because the “resident” tetramers are held under tension in a fully-stretched conformation, energetically unfavorable for a free tetramer (Lai and Cao, 2014; Zhang et al., 2017).

## The MPS and Axonal Stability

It was initially suggested that the MPS could be necessary for the structural stability of the axon as it may provide distinctive mechanical properties. However, the structural consequences of MPS disassembly are not that easy to grasp. For instance, actin-destabilizing regimes that acutely deplete axons from MPS are not accompanied by axon destruction (Zhong et al., 2014; Valakh et al., 2015; Qu et al., 2017). Indeed, reports on the systematic deletion of structural components of the MPS or enzymes related to actin polymerization in *Drosophila melanogaster* reported no evidence of axon destruction (Qu et al., 2017). This evidence suggests that the MPS is not essential for the structural stability of the axon in the basal state.

However, axons innervating moving parts of the body of *Caenorhabditis elegans* or zebra fish depleted from βII-spectrin are damaged by physical stress associated with animal movement (Hammarlund et al., 2007; Krieg et al., 2017). A recent study has evaluated the relationship between the MPS and axon stability in a more physiological setting. Developmental axon pruning can be modeled in cell culture by withdrawing dorsal root ganglion explants from Nerve Growth Factor (Glebova and Ginty, 2005). Under this condition, the axonal MPS shows a significant decrease in abundance during most of the degeneration period, and is fully dismantled in fragmenting axons. Interestingly, acute pharmacological stabilization of F-actin prevents both MPS loss and axonal fragmentation (Unsain et al., 2018). Taken together, these studies suggest that the MPS is not required to keep axon integrity under basal conditions, but instead it might be necessary for coping with physical stress or degenerative triggers.

From a structural perspective, it is yet not clear how the MPS and microtubules (MT) work together to provide the extraordinary mechanical properties of the axon. Small molecule-induced depolymerization approaches have highlighted their inter-dependance. In *Drosophila* neurons, disruption of the MPS with low doses of cytochalasin D affects MT polymerization, ultimately leading to MT “gaps” (Qu et al., 2017). In mouse neurons, deletion of α-adducin affects MT-based transport (Leite et al., 2016). Also, the concomitant disruption of the actin cytoskeleton and the deletion of the MT stabilizing protein Shot induce axonal loss. Conversely, MT depolymerization induced by nocodazole disrupts the MPS (Zhong et al., 2014; Qu et al., 2017). Additional evidence for an interaction between MT and the MPS comes from the observation that MPS-bound ankyrin G regulates MT dynamics, protein transport and AIS stability/maintenance through physical interactions with EB1 and EB3 at their deep C-terminus tail (Leterrier et al., 2011, 2015) or by functional interaction with CRMPs (Maniar et al., 2011).

The MPS would allow the axon membrane to sustain shear and torsional efforts, since membrane stiffness measurements with atomic force microscopy (AFM) have established that the axon has a superior resistance than the somato-dendritic compartment (Zhang et al., 2017). Also, it has been proposed that myosin-dependent contraction of actin rings, counteracted by the underlying MT bundle, would induce a torsional tension necessary to maintain axon volume in response to external stretch or compression forces (Fan et al., 2017). Direct evidence that actin rings may function as a contractile actomyosin complex comes from the finding of a periodical co-localization of phosphorylated myosin light chain (pMLC) with actin at the AIS-MPS (Berger et al., 2018).

## Plasma Membrane Biology

The EMCC effectively confines the movement of peripheral or integral membrane proteins in erythrocyte (Tomishige et al., 1998). The importance of this function is highlighted by hereditary pathologies showing lack of confinement of erythrocyte membrane proteins (Kodippili et al., 2009). Confined phospholipid diffusion by the underlying actin/spectrin cortical skeleton was also recently observed in other cell types using super-resolution microscopy (Andrade et al., 2015). Also, it has been found that membrane-bound probes in the AIS follow transversally oriented trajectories confined to *stripes* spaced by 190 nm. These trajectories co-localize with the spectrin of the MPS, with the underlying *actin stripes* serving as the confinement “fence” (Albrecht et al., 2016). Interestingly, the authors failed to observe such motion confinement outside the AIS, suggesting that AIS-exclusive protein/s organized by the MPS but not the MPS itself is/are responsible for the effective confinement of lateral diffusion (see Huang and Rasband, 2016, for a review). A non-excluding possibility is that the probes were actively anchored by spectrin related components specific of the AIS.

Extensive evidence supports a critical role of the actin/spectrin skeleton in ligand-receptor signaling, membrane budding and endocytosis. However, it is still unknown how these functions are affected when this cortical network is organized in periodical stripes, which likely produce parallel equivalent subdomains of membrane proteins and/or spatio-temporal structures key for signaling (Grecco et al., 2011).

## Concluding Remarks

Current challenges concerning the MPS can be divided in two main aspects. First, its detailed structure must be determined, including the molecular interactions that hold it together and linked to the plasma membrane. For instance, Band 3 is a transmembrane protein that is key to tether the EMCC to the plasma membrane. In neurons, however, Band 3 is not expressed and it is still unknown which proteins, if any, play an equivalent role. Second, the biological functions of the MPS remain uncertain, both in terms of structure and mechanical properties, as well as signaling, including action potential propagation, and cell-cell interactions.

To assess these questions, it will be critical to develop approaches to specifically disrupt the MPS structure without affecting the behavior of its components outside of the MPS. For instance, the use of F-actin depolymerizing drugs to disassemble the MPS also affects actin filaments elsewhere in the cell, and hence conclusions regarding the MPS are limited. The same drawback can be said when evaluating the deletion of spectrins.

Another challenge is to understand what are (if any) the functional consequences of a periodicity in the longitudinal aspect of an axon, both structurally and functionally. For instance, the membrane stripes organized by the underlying MPS would likely confine periodically ion channels in the longitudinal axis of the axon, which may impact, or actually be crucial for proper action potential propagation.

In summary, the recent discovery of the MPS has created new, exciting avenues of research that are just beginning to be explored and will likely provide solid ground to tackle long-lasting questions of neurobiology.

## Author Contributions

NU, FS and AC wrote and revised the manuscript.

## Conflict of Interest Statement

The authors declare that the research was conducted in the absence of any commercial or financial relationships that could be construed as a potential conflict of interest.
